# Individual differences in regulatory mode moderate the effectiveness of a pilot mHealth trial for diabetes management among older veterans

**DOI:** 10.1371/journal.pone.0192807

**Published:** 2018-03-07

**Authors:** Michelle Dugas, Kenyon Crowley, Guodong Gordon Gao, Timothy Xu, Ritu Agarwal, Arie W. Kruglanski, Nanette Steinle

**Affiliations:** 1 Department of Psychology, University of Maryland, College Park, Maryland, United States of America; 2 Center for Health Information & Decision Systems, Robert H Smith School of Business, University of Maryland, College Park, Maryland, United States of America; 3 College of Information Studies, University of Maryland, College Park, Maryland, United States of America; 4 Decision, Operations, & Information Technologies, Robert H Smith School of Business, University of Maryland, College Park, Maryland, United States of America; 5 Department of Biology, Emory University, Atlanta, Georgia, United States of America; 6 Maryland Veterans Administration Health Care Center, Baltimore, Maryland, United States of America; 7 University of Maryland School of Medicine, Baltimore, Maryland, United States of America; Florida International University Herbert Wertheim College of Medicine, UNITED STATES

## Abstract

mHealth tools to help people manage chronic illnesses have surged in popularity, but evidence of their effectiveness remains mixed. The aim of this study was to address a gap in the mHealth and health psychology literatures by investigating how individual differences in psychological traits are associated with mHealth effectiveness. Drawing from regulatory mode theory, we tested the role of locomotion and assessment in explaining why mHealth tools are effective for some but not everyone. A 13-week pilot study investigated the effectiveness of an mHealth app in improving health behaviors among older veterans (*n* = 27) with poorly controlled Type 2 diabetes. We developed a gamified mHealth tool (DiaSocial) aimed at encouraging tracking of glucose control, exercise, nutrition, and medication adherence. Important individual differences in longitudinal trends of adherence, operationalized as points earned for healthy behavior, over the course of the 13-week study period were found. Specifically, low locomotion was associated with unchanging levels of adherence during the course of the study. In contrast, high locomotion was associated with generally stronger adherence although it exhibited a quadratic longitudinal trend. In addition, high assessment was associated with a marginal, positive trend in adherence over time while low assessment was associated with a marginal, negative trend. Next, we examined the relationship between greater adherence and improved clinical outcomes, finding that greater adherence was associated with greater reductions in glycated hemoglobin (HbA1c) levels. Findings from the pilot study suggest that mHealth technologies can help older adults improve their diabetes management, but a “one size fits all” approach may yield suboptimal outcomes.

## Introduction

The number of American adults with diabetes has quadrupled since 1980 and associated costs have reached over 200 billion dollars annually[1], leaving both patients and health care workers in search of ways to improve diabetes management. Although everyday decisions about eating habits, exercise, and medication adherence are critical to controlling progression of the disease, many patients report barriers to enacting these behaviors[2,3]. Mobile health (mHealth) technologies use mobile and wireless devices to improve health, and are viewed as a promising medium to help patients overcome barriers and achieve their health goals[4,5], but most existing studies focus on average treatment effects that may not be experienced uniformly across target populations. Indeed, theories from social and personality psychology suggest that health interventions might provide a better fit for some individuals than others[6,7], offering potential insights into heterogeneous mHealth effects. Thus, the objective of the present pilot research was to explore the interplay of individual differences in regulatory mode and mHealth in motivating lifestyle change among older veterans, a population experiencing a heavy diabetes burden that is also underrepresented in the mHealth intervention literature.

### Diabetes in older adults and veterans

Rates of Type 2 diabetes in older adults are higher than other populations, with approximately 20% of Americans over the age of 65 suffering from diabetes[1]; and consequences of the disease can be especially serious including heightened mortality and reduced functionality[8]. Despite greater disease prevalence and associated risks among older adults, they are underrepresented in controlled trials designed to improve diabetes management[9,10]. Veterans are another large population at high risk of diabetes and its complications, and older veterans often have worse health outcomes than older adults in the general population[11]. Concerns related to diabetes self-management are exacerbated by the fact that primary care providers, who typically manage diabetes in the initial stages of disease, do not devote sufficient time to diabetes management[12]. In resident-staffed general medicine clinics, residents spent an average of 5 out of 25 minutes on diabetes, and evaluation of glycated hemoglobin (HbA1c) levels were addressed only 40% of the time[12].

Given the time constraints experienced by health care providers and growing prevalence of diabetes in the U.S., it is imperative that persons with diabetes are equipped with tools that raise awareness of the impact of their lifestyle choices and motivate them to better self-manage their diabetes. Moreover, it is equally critical that we find ways of identifying who tends to benefit most from different types of self-management interventions to ensure that patients are provided with care that matches their needs and preferences. With the unique health needs of veterans and the underrepresentation of older adults in diabetes interventions research considered, we sought to explore the role of theoretically relevant psychological traits in moderating effectiveness of an mHealth app promoting better management of diabetes.

### mHealth and chronic disease management

Recent polls show that a majority of US adults own a smartphone[13], and interest in mHealth technologies is not restricted to the young—most older adults report being eager to adopt mobile fitness technologies[14,15]. mHealth technologies offer many advantages that are appealing for interventions including their widespread accessibility, cost-effective delivery, and flexibility to content tailoring[4]. These benefits of mHealth technologies have led to a surge in their use to address a major health challenge—chronic disease self-management. Although there is encouraging evidence of success[16], several reviews of the effectiveness of mHealth interventions have yielded mixed results for treatment adherence[17,18] and clinical outcomes[19,20].

Inconsistencies in findings highlight some of the challenges endemic to longitudinal mHealth interventions including high drop-out rates and weakened engagement over time[21,22]. While these problems have often been noted as barriers to maximizing the impact of mHealth initiatives, theory-based approaches to understanding who will benefit from such interventions have been underutilized. To address this gap, we drew on insights from the psychology of motivation to explore individual differences that could explain heterogeneity in the effectiveness of an mHealth intervention to improve diabetes self-management.

### Individual differences and mHealth effectiveness

Even as the use of mHealth in interventions has surged, its integration with health and personality psychology is nascent[23]. Illustrating this point are content analyses of mHealth applications that have shown low integration of apps with health behavior theory[24,25]. One particularly important connection between theory and mHealth could lie in understanding the role of personality in shaping engagement with health interventions[26]. The present research offers an exploration of this connection by focusing on a personality dimension implicated in motivation that could moderate the effectiveness of our mHealth intervention, viz., regulatory mode.

#### Regulatory mode

*Regulatory mode theory* posits that two independent orientations underlie most self-regulation, locomotion and assessment[27,28]. Locomotion refers to a preference for movement from state to state and is captured by the phrase “just do it.” Assessment, on the other hand, reflects a preference for evaluating states and alternatives and can be characterized by the phrase “do the *right* thing.” The two dimensions of regulatory mode are orthogonal[29] and differentially related to a wide range of phenomena including regret[30], burnout[31,32], and risk-taking[33].

We propose that regulatory mode orientations may influence effectiveness of interventions that are centered around goal-setting and self-monitoring, as in the case of our mHealth application DiaSocial, developed internally by the research team for the study. Locomotors, in particular, might benefit from an mHealth app’s role in providing patients with specific, salient health behavior goals through features like gamification. Gamification is a term that refers to integrating game mechanisms into non-game contexts, such as using leaderboards and point systems that reward certain behavior[34]. A gamification system that outlines goals for various health behaviors could instigate behavior change in locomotors who tend to act on goals efficiently[35] and with little procrastination[36]. In accordance with this reasoning, we expect our mHealth intervention to be especially effective for high (vs. low) locomotors, as they should be more eager to act on the goals provided by the gamification point system.

We also expect assessors to benefit from the intervention, although through a different mechanism, which is self-monitoring. Self-monitoring is considered important in the management of chronic diseases[37,38], and many mHealth tools endeavor to facilitate tracking of health behavior[39,40]. However, self-monitoring with mHealth tools often requires some component of manual data entry, imposing the burden of substantial effort and non-trivial demands on patients. Accordingly, mHealth tracking tools may not be equally appealing to everyone. Given assessors’ preference for comparison and self-evaluation, we predict that high (vs. low) assessment will be positively associated with engagement with an mHealth tool and sustained behavior change over the course of an intervention, as indicated by self-reported treatment adherence. In other words, we expect the emphasis on self-monitoring to “fit” with assessors’ orientation towards evaluation, increasing engagement with the app, thereby improving diabetes outcomes[41].

### The present research

The present research explored the utility of the mHealth tool described above, the DiaSocial app, in improving diabetes outcomes in a sample of older veterans. A central objective of our pilot was to explore whether individual differences in regulatory mode moderated the effectiveness of our mHealth intervention in increasing healthy behavior and improving clinical outcomes. We predicted that locomotion and assessment would both independently moderate the effectiveness of the app due to different mechanisms. More specifically, we expected the gamification features to be particularly motivating to high (vs. low) locomotors who are eager to act on salient goals, resulting in greater adherence. Similarly, we expected the tracking features of the app would appeal to those high (vs. low) in assessment, motivating treatment adherence. In turn, we expected that greater levels of adherence would be associated with better clinical outcomes.

## Methods

### Participants

**Fig 1:**
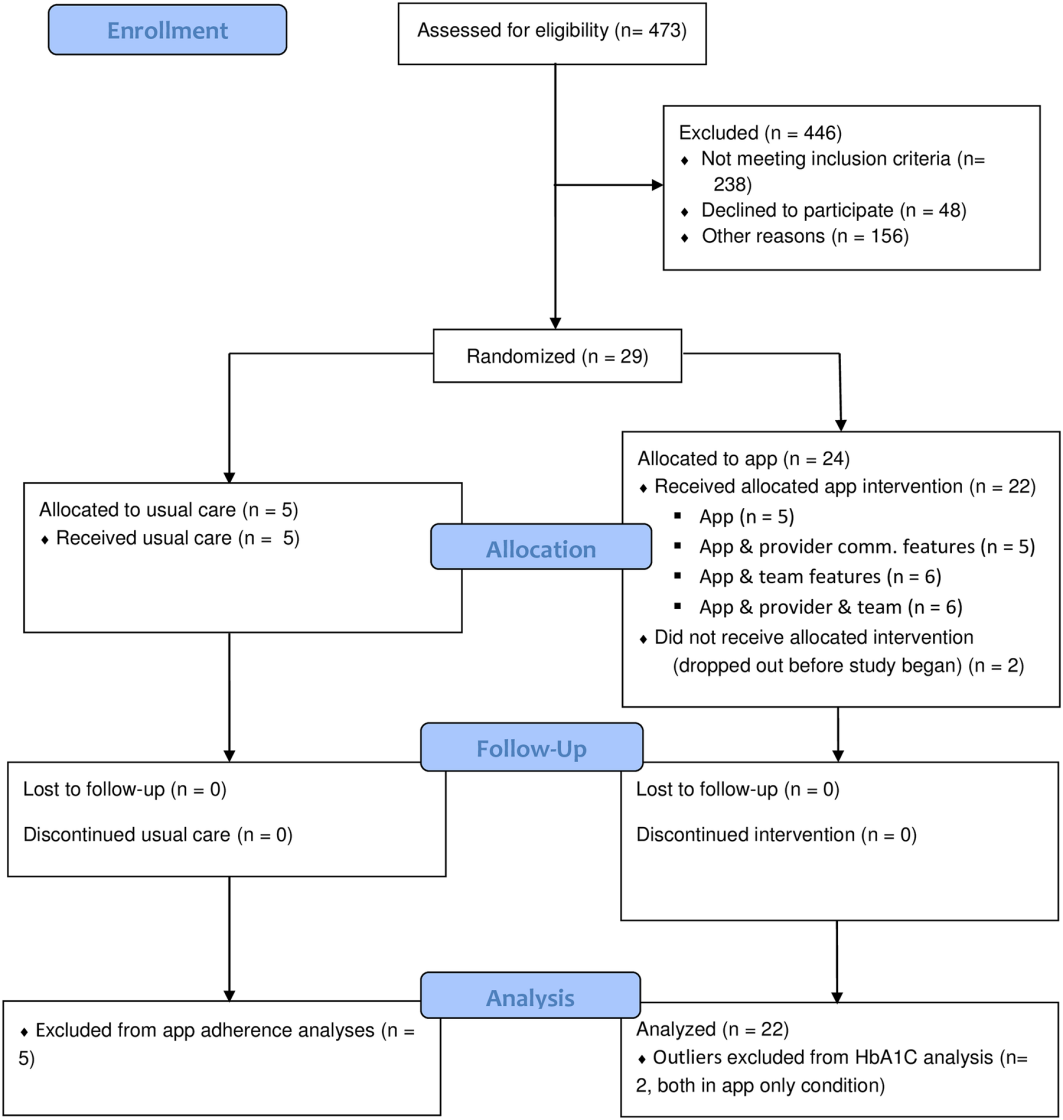
CONSORT participant flow diagram.

All research participants were veteran patients affiliated with an endocrinology clinic of a VA medical center. Patients aged 60 and above having a known diagnosis of Type 2 diabetes for >3 years, and poorly controlled (HbA1c > 7.9%) were eligible to participate. Exclusion criteria included blindness, deafness, a diagnosis of serious mental illness (active psychosis, bipolar disorder, schizophrenia, borderline personality disorder, active alcohol or other substance misuses) and homelessness. A study signup flyer was posted in the clinic waiting room and staff shared the study participation opportunity with eligible patients. Seventy-nine patients expressed initial interest in the study and were sent letters detailing the study and formally inviting them to participate. Thirty-one patients responded to the letters, two withdrew before the beginning of the study period, leaving a final sample of twenty-nine research participants aged 61–86, including three women, and three who self-identified as African American. Two additional recruits dropped out shortly after study initiation, leaving 27 total participants (*M*_age_ = 67.56, *SD*_age_ = 5.81). Participant exclusions and drop-outs are summarized in Fig 1 and additional detail is presented in the CONSORT checklist in S1 File. Initially, we sought to recruit about 88 participants, but our final sample size was limited by funding for the project, which came internally from the research institutions of a subset of the authors and the Theo und Friedl Schöller Foundation.

Participants received a Samsung Galaxy Tab 3 with data plan, a Fitbit One, as well as $20 in travel support for each of the clinic visits. The participants were allowed to keep the devices at study end. The study was reviewed and approved by the University of Maryland Baltimore IRB, and all participants provided signed informed consent. The study was also registered on ClinicalTrials.gov (#NCT02127216).

### Procedure

The study protocol is available as supplementary information in S2 File. Participants were enrolled and completed an initial questionnaire including demographic information and a personality inventory during their regular diabetes clinic visit. Participants were then block randomized into usual care or one of four experimental conditions, with patients stratified by age, baseline HbA1C, and BMI. At study outset, participants completed a 2-hour group training session where they were given a Samsung Galaxy Tab 3 preloaded with the DiaSocial app, configured to match their experimental group (i.e., team grouping, provider communication), and a Fitbit One. Operation of the tablet, DiaSocial app and Fitbit were demonstrated, and participant questions were addressed. Individuals were instructed to use the app daily, and were encouraged to earn points by recording their progress in achieving better diabetes self-care goals through managing glucose level, exercise, diet, and medication adherence. Participants who were assigned to teams met their teammates in person during the training session and were able to continue to interact online through the app.

The treatment period began the Monday following training, which occurred during February, and lasted for 13 weeks to assess trends in healthy behavior (as assessed by gamification points earned through the app) and changes in HbA1C. The duration of the intervention was constrained by available resources for the pilot study, therefore 13 weeks was selected because it represented the shortest amount of time at which reliable changes in our primary clinical outcome of interest, HbA1C, could be observed.

#### Manipulations

In order to pilot test different mHealth engagement strategies, a parallel research design with five conditions was employed. The research participants were assigned to a usual care group (*n* = 5) or one of four experimental conditions: (T1) Patients using the app individually without clinician or peer engagement (*n* = 5); (T2) Patients using the app with clinician engagement features (*n* = 5); (T3) Patients using the app with peer engagement features (*n* = 6), and (T4) Patients using the app with both clinician engagement and peer engagement features (*n* = 6). Participants were randomly assigned to conditions using the blockrand package in R with a block size of 6 and stratifying participants across four blocking factors (age, gender, HbA1C, and BMI). Neither patients nor clinicians were blind to treatment conditions. A summary of descriptive statistics by experimental conditions can be found in Table 1.

**Table 1:** Summary statistics by condition for enrolled sample.

	Control		T1		T2		T3		T4	
	(n = 5)		(n = 5)		(n = 5)		(n = 6)		(n = 6)	
	*M*	*SD*	*M*	*SD*	*M*	*SD*	*M*	*SD*	*M*	*SD*
Age	66.40	4.93	65.40	4.72	72.00	9.30	66.00	5.18	68.17	3.66
Locomotion	3.30	0.36	3.67	0.88	3.83	0.47	3.89	0.51	3.61	0.43
Assessment	2.83	0.26	2.86	0.65	2.83	0.94	2.78	0.83	2.88	0.50
Days App Used	--	--	61.20	21.87	67.80	20.98	61.67	30.69	69.50	31.15
Total Score	--	--	4436.60	3037.35	4326.40	1815.09	4360.00	2750.48	5173.33	3055.62
Pre-A1C	9.12	0.64	9.44	1.42	9.34	0.93	8.47	0.66	8.66	.36
Post-A1C	8.78	0.89	10.14	3.09	8.96	0.55	8.36	0.60	8.06	.78

Patients in all four experimental conditions received the same app self-monitoring features. Patients were able to view if their scores were trending at, above, or below the point goals for each behavior. Provider engagement features allowed a clinician to view a patient’s behavior score trends and raw data (e.g. glucose reading or minutes exercised) and communicate with the patient. Similarly, team features allowed for communication between team members and allowed team members to view each other’s progress on daily and weekly points, and their team’s progress in relation to another team.

### Measures

#### Regulatory mode

Regulatory mode was assessed with adapted 6-item brief versions of the locomotion (e.g., “I enjoy actively doing things, more than just watching and observing”; α = .60) and assessment scales (e.g., “I spend a great deal of time thinking about my positive and negative characteristics”; α = .70)[28]. Response categories ranged from 1 (*Strongly Disagree*) to 5 (*Strongly Agree*), and average scores for each scale were used for analyses.

#### HbA1C

Glycated hemoglobin (HbA1C) measures were taken as our key clinical outcome given that higher HbA1C measures have been associated with greater diabetes complications and poorer health outcomes, and controlling HbA1C is a primary therapeutic target[42]. HbA1C levels were assessed via a blood draw and laboratory testing at the VA clinical laboratory. If patients had their HbA1C levels assessed within 3 months of being recruited and enrolled in the study, this was treated as their baseline measure to avoid undue burden from another blood test. We then aimed to assess post-study HbA1C levels 90 days after the beginning study. Given differences in recruitment times and regular clinic appointments for each patient, however, there was variability in the dates at which HbA1C levels were measured (S1 Appendix for details).

#### Adherence

Health behaviors related to diabetes management were allocated daily points when reported in the app, and these points were used to assess treatment adherence during the intervention. Points were allocated for achieving daily goals related to reporting and reaching target levels of glucose, exercise, nutrition, and medication adherence (S1 Appendix). Glucose, diet, and medication tracking were entered manually by participants, but exercise tracking could be synchronized with their Fitbit or entered manually. Scores across different domains of adherence were significantly correlated with each other and therefore scores were summed together to form an overall adherence score. Daily overall adherence scores were then summed to form weekly scores and one total score representing adherence over the course of the 13-week study. Each participant’s individual weekly scores are presented by domain in S1 Appendix.

## Statistical method

When designing our pilot study, we were primarily interested in how individual differences could moderate the impact of an mHealth intervention with a focus on regulatory mode dimensions of locomotion and assessment. However, our research design also included several treatment conditions to explore the feasibility and appeal of different app features (e.g., teams, provider communication). As such, we conducted one set of analyses to explore how different treatments impacted the effectiveness of the mHealth app and a second set of analyses that focused on the role of regulatory mode. One-way ANCOVA and linear mixed effects regression models were used to explore these research questions and were performed with IBM SPSS 24 software[43]. Given the exploratory nature of our pilot study, we did not control for multiple comparisons and set a threshold of significance at *p* = .05 for each test.

First, we explored between-group treatment effects on overall adherence and HbA1C in our analyses. We did not expect strong between-group effects given some of the limitations in the piloted communication features and the small sample sizes per group. Therefore, to explore treatment effects on adherence, we performed a one-way ANCOVA testing mean differences in total adherence during the study. Next, we tested for interactions of treatment effect and time on HbA1C. If treatment conditions interacted with time, then this would constitute evidence that the app features affected the degree of change in an important clinical outcome.

After exploring between-group differences, we then turned to testing the hypothesized role of regulatory mode in promoting greater self-management adherence over the course of the 13-week intervention. Debriefing interviews revealed that participants did not fully utilize communication features of the app, perhaps because this aspect of the software was still in need of some refinement. Given that the communication features were not fully utilized, small sample sizes per condition (*n* < 7), and mostly null differences between treatment groups, we examined the role of regulatory mode among participants assigned to the experimental arms of the study independent of treatment condition, simply controlling for assignment to different study arms rather than exploring possible interaction effects of treatment group and regulatory mode.

We were particularly interested in exploring how regulatory mode affected not only overall adherence throughout the study, but also trends in weekly adherence. With engagement and behavior change maintenance being a concern in mHealth interventions[21,22], we expected that weekly adherence could exhibit a curvilinear trend over the course of the intervention’s 13 weeks. Specifically, we thought it plausible that patients would show an initial upwards trend in adherence behavior as they built off the excitement of a new intervention and any early successes. However, we recognized that the novelty of the intervention could begin to wane as participants progressed through the later stages of the intervention, as has been observed in the case of other mHealth interventions. Moreover, a plot of the observed levels of average adherence at each week suggested that a quadratic trend might better approximate the pattern of adherence over time than a linear trend (see S1 Appendix Therefore, we explored both linear and quadratic effects of time in our analyses with regulatory mode. We hypothesized that locomotion and assessment would each interact with a quadratic effect of time such that locomotors and assessors were better able to maintain their strong adherence over the course of the 13-week duration of the study.

After testing for the hypothesized role of regulatory mode on adherence, we sought to explore whether measures of adherence were associated with improvements in our clinical outcome, HbA1C. Such a relationship would provide initial support for the validity of our gamification point structure and would illustrate the utility of identifying personality characteristics associated with greater adherence. Thus, we examined the interaction effect of total adherence score and time to predict HbA1C levels. We expected an interaction effect such that a greater drop in HbA1C levels is observed among those who scored high on measures of adherence.

## Between-group results

### Adherence

Before proceeding with inferential tests, we first created a boxplot to explore the distribution of adherence scores to identify possible outliers (see S1 Appendix). Satisfied with the distribution of our outcome variable, we proceeded to conduct a one-way ANCOVA, including a treatment factor of the four treatment arms of the study and age as a covariate. The control group was not included in these analyses because adherence was assessed with points earned through the app. Age was unrelated to total adherence(*F*(1, 17) = -0.34, *p* = .5, *η*_*p*_^2^ = .02). In addition, there was a non-significant treatment effect, *F*(3, 17) = 0.14, *p* = .93, *η*_*p*_^2^ = .02. Table 1 summarizes the differences in total adherence between treatment groups. As expected, there were no differences in adherence levels across treatment conditions.

### Changes in HbA1C

We again plotted boxplots to explore the distribution of HbA1C to identify possible outliers. Two individuals were identified as having outlier HbA1C levels (see S1 Appendix) and were excluded from further analyses, yielding a final sample size of 25. A linear mixed effects model was then estimated to test for differences between treatment conditions in the rate of change of HbA1C from baseline to post-intervention. Model specifications are further detailed in S2 Appendix.

As evident from Table 2, between-group comparisons detected no significant effects on HbA1C change over time. As noted earlier, however, we did not expect between-group differences to emerge given the relatively small sample sizes per condition. Moreover, we were particularly interested in examining the effects of regulatory mode as a moderator of the effectiveness of an mHealth intervention. In the proceeding analyses, we explore the role of individual differences in regulatory mode in adherence and, in turn, the role of adherence in yielding greater clinical benefits from an intervention.

**Table 2:** Model estimates predicting HbA1C.

	Est.	SE	*t*	*p*
*Fixed Effects*				
Intercept	9.07	0.35	26.21	< .001
Age	-0.03	0.02	-1.20	.24
Time	-0.34	0.39	-0.88	0.39
T1	-0.38	0.56	-0.68	.50
T2	0.38	0.50	0.75	0.46
T3	-0.66	0.46	-1.43	.16
T4	-.0.37	0.47	-0.79	.43
Time x T1	-0.49	0.64	-0.78	.45
Time x T2	-0.04	0.55	-0.07	.94
Time x T3	0.31	0.53	0.59	.56
Time x T4	-0.49	0.53	-0.93	.36
*Random Effects*				
σ^2^	0.38	0.12		< .01
τ_00_	0.21	0.14		.14

σ^2^ = residual, τ_00_ = variance in intercept by participant.

## Individual difference results

### Adherence

In our pilot study, we were particularly interested in exploring the moderating role of regulatory mode on the effectiveness of an mHealth intervention. Based on hypotheses derived from regulatory mode theory, we expected trends in weekly adherence scores to depend on locomotion and assessment as locomotors would benefit from the goal-directing aspects of the app while assessors would benefit from the app’s facilitation of self-monitoring. As such, we hypothesized that adherence would, on average, exhibit a downward quadratic trend, but that the negative trend in the second half of the study would be attenuated by high locomotion and high assessment. To this end, we introduced locomotion and assessment as predictors of adherence scores and tested potential cross-level interaction effects with the linear and quadratic effects of time. Model specification details are reported in S2 Appendix. Analyses pertaining to specific adherence domains (glucose, nutrition, exercise, and medication) can also be found in S2 Appendix.

**Fig 2:**
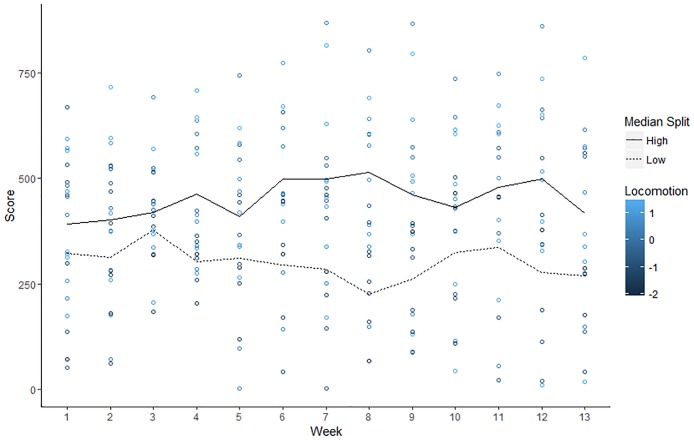
Observed values of for weekly adherence scores by individual level of locomotion. Colors represent standardized locomotion scores of participants and lines show average weekly score for low (< 3.67 scale score) and high (> 3.67 scale score) locomotors as defined by a median split.

**Fig 3:**
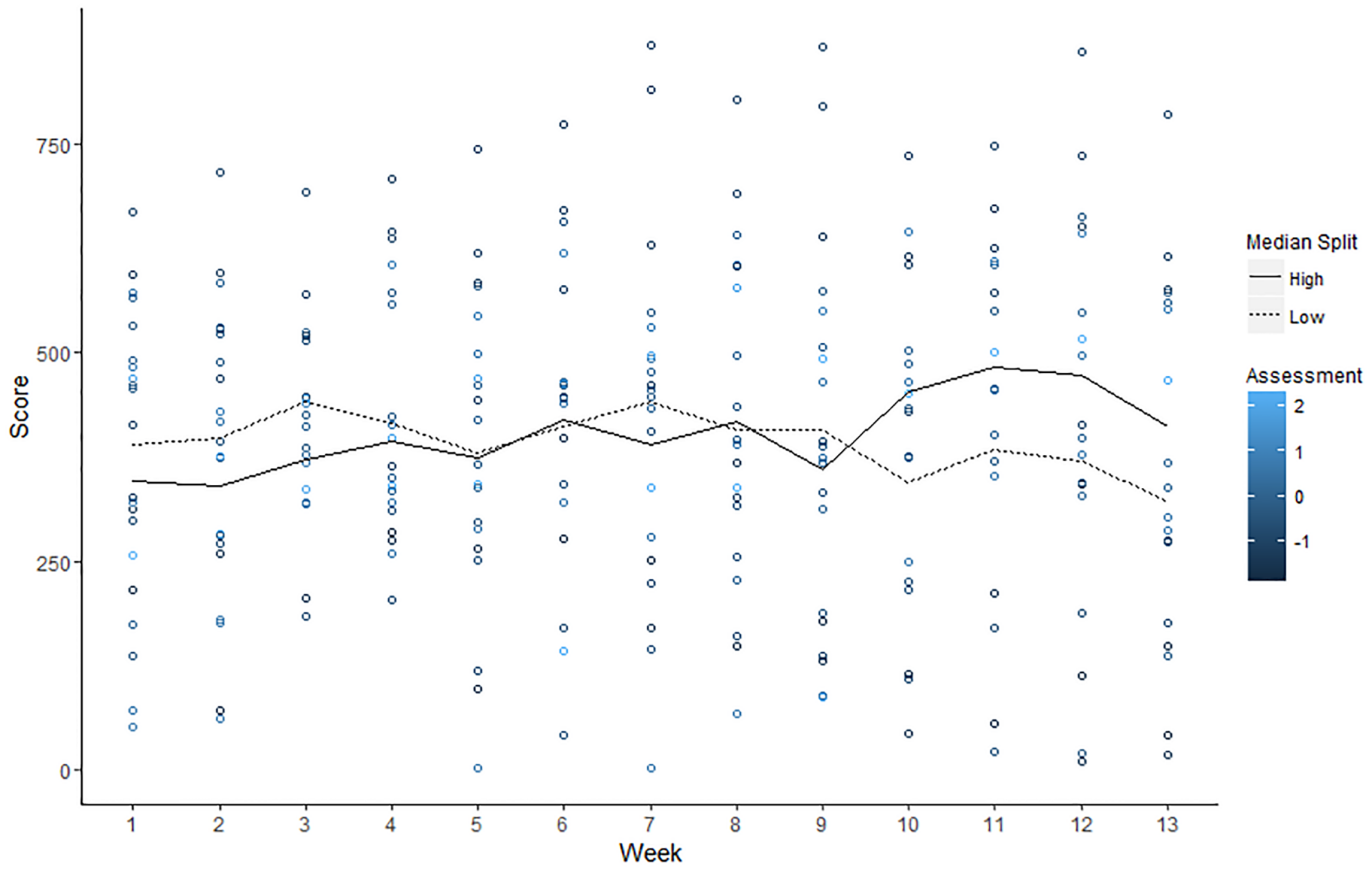
Observed values of for weekly adherence scores by individual level of assessment. Colors represent standardized locomotion scores of participants and lines show average weekly score for low (< 2.83 scale score) and high (> 2.83 scale score) assessors as defined by a median split.

Relevant descriptive statistics are reported in Table 3. Observed trends in adherence for individuals high and low in locomotion and assessment are illustrated with Figs 2 and 3, respectively.

**Table 3:** Descriptive statistics and Bivariate correlations (N = 22).

	*M*	*SD*	1	2	3	4
1. Age	67.82	6.07	--			
2. Locomotion	3.79	0.50	-.44*			
3. Assessment	2.84	0.63	.06	.06		
4. Days App Used	65.09	25.38	.06	.32	.16	
5. Total Adherence Score	4591.59	2558.03	-.13	.41^ǂ^	.03	.85***

^ǂ^ *p* < .10.

*p < .05.

*** *p* < .001.

**Fig 4:**
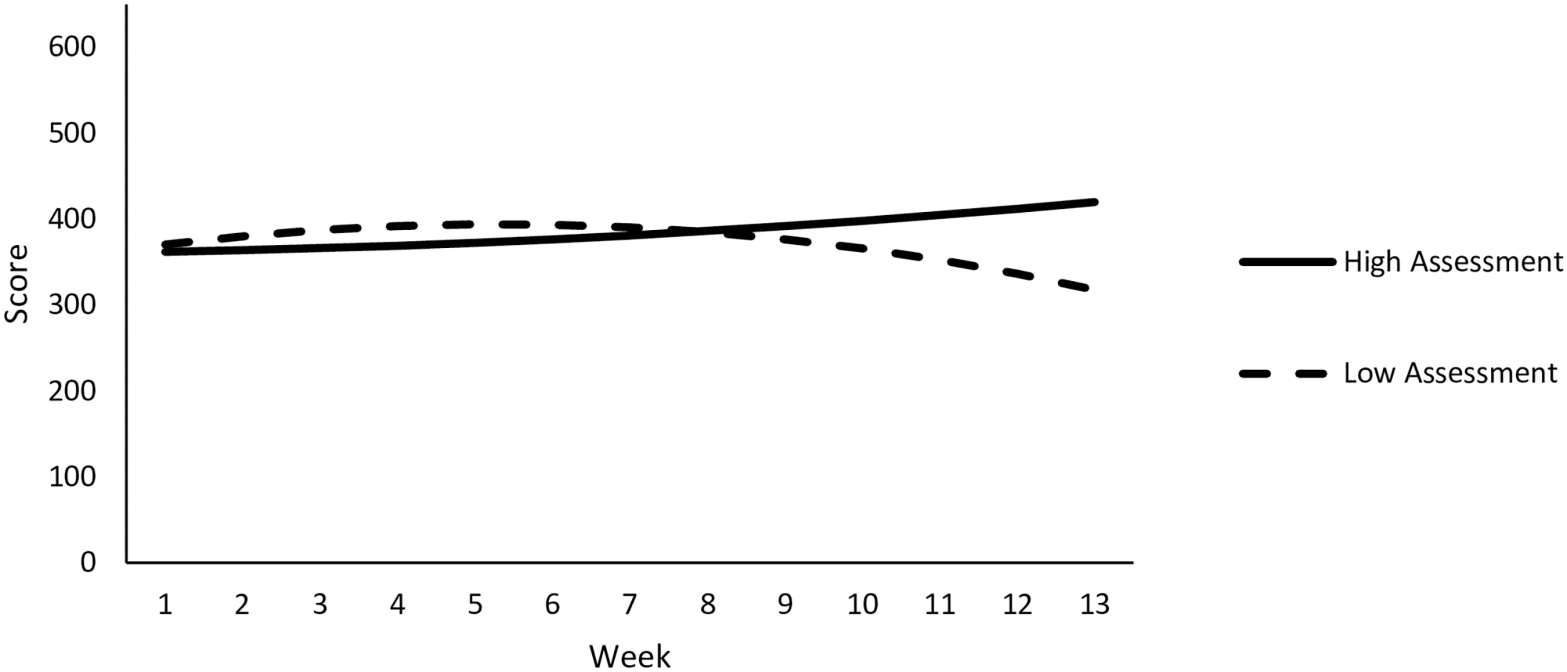
Patterns of predicted weekly adherence scores over time at high and low levels of assessment are depicted.

**Fig 5:**
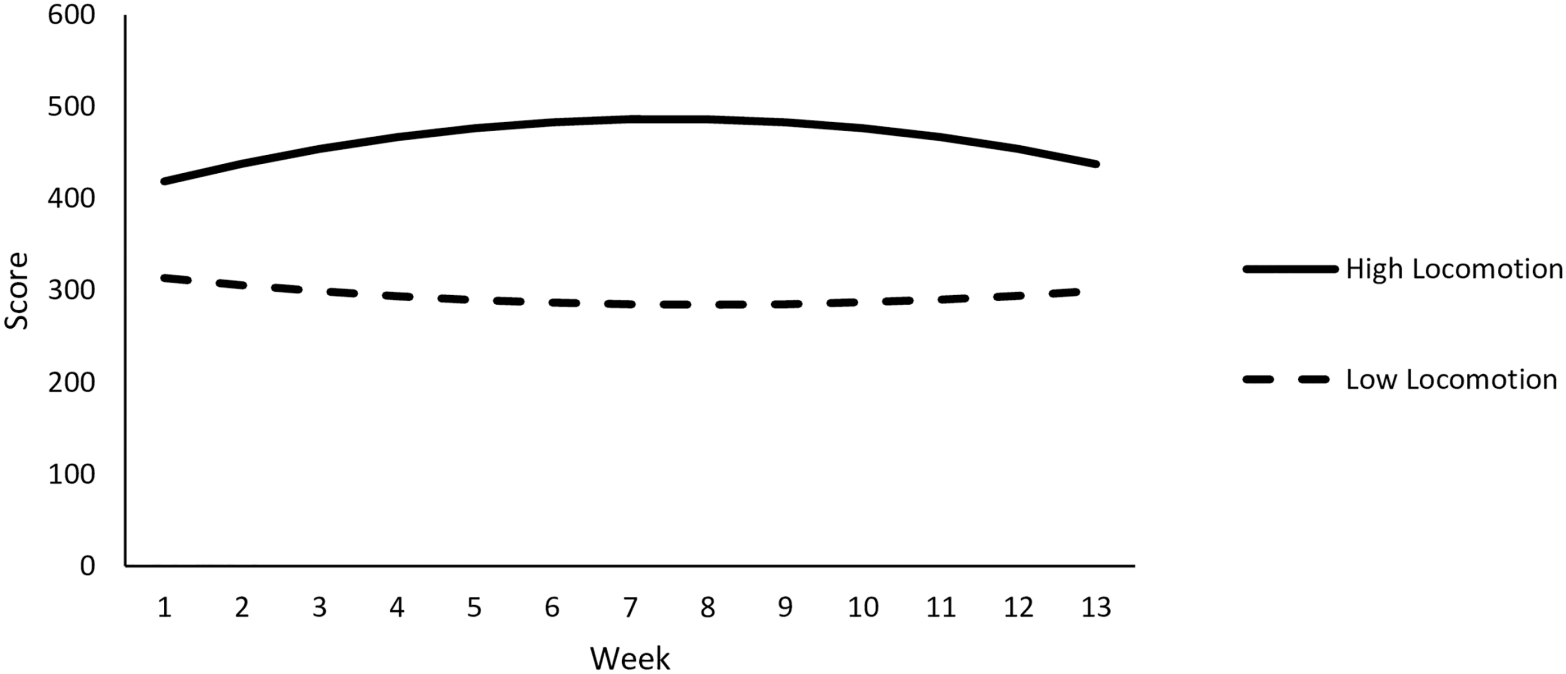
Patterns of predicted weekly adherence scores over time at high and low levels of locomotion are depicted.

Results of linear mixed models are summarized in Table 4 (and a plot of residual vs. predicted values is reported in S1 Appendix). Notably, there was a non-significant linear effect of time on adherence, B = .22, *t*(224.42) = 0.12, *p* = .90. In addition, there was a non-significant of quadratic effect of time, B = -0.51, *t*(223.68) = -1.96, *p* = .34. However, these effects were qualified by significant interactions. First, there was a significant interaction between the linear trend in time and assessment, B = 7.31, *t*(226.59) = 2.50, *p* = .01. Furthermore, there was a significant interaction effect of the quadratic term for time and locomotion, B = -2.13, *t*(223.78) = - 2.04, *p* < .05. In light of the significant interaction effects, we performed conditional analyses to examine the linear trend in adherence at low (-1 SD) and high (+1 SD) levels of assessment (Fig 4) and the quadratic trend in adherence at low (-1 SD) and high (+1 SD) levels of locomotion (Fig 5).

**Table 4:** Model estimates predicting weekly adherence scores.

	Est.	SE	*t*	*p*
*Fixed Effects*				
Intercept	383.12	38.15	10.04	< .001
Age	-.09	7.95	-0.01	.13
T2	-49.02	74.36	-0.66	.52
T3	4.74	64.25	0.07	.94
T4	60.67	64.68	0.94	.36
Time	0.22	1.81	0.12	.90
Locomotion	193.64	90.90	2.13	< .05
Assessment	-7.37	57.39	-0.13	.90
Time^2^	-0.51	.53	-0.96	.34
Time x Locomotion	2.61	3.53	0.74	.46
Time x Assessment	7.31	2.92	2.50	.01
Time^2^ x Locomotion	-2.13	1.05	-2.04	.04
Time^2^ x Assessment	1.24	0.83	1.49	.14
*Random Effects*				
σ^2^	10553.13	999.71		< .001
τ_00_	29182.90	11134.59		< .01

σ^2^ = residual, τ_00_ = variance in intercept by participant.

**Assessment**. Conditional analyses decomposing the Time x Assessment interaction effect found a marginal decrease in adherence over time at low levels of assessment, B = -4.39, *t*(226.52) = -1.73, *p* = .08. In contrast, there was a marginal increase in adherence over time at high levels of assessment, B = 4.82, *t*(224.71) = 1.83, *p* = .07. Controlling for individual locomotion, treatment condition, and age, these results suggest that individuals higher in assessment might benefit from a self-monitoring oriented mHealth intervention to a greater extent than individuals low in assessment. However, the marginal significance of our simple slope analyses underscores the need for caution in interpretation of these findings, and reveal a need for further research into this question. This is especially important given that the plot depicting the association between observed scores of assessment and adherence did not reveal a strong relationship (Fig 3).

**Locomotion**. Next, we proceeded to decompose the Time^2^ x locomotion interaction with conditional analyses. A non-significant quadratic effect of time on adherence scores at low levels of locomotion was found, B = 0.60, *t*(224.34) = 0.75, *p* = .45. In contrast, there was a significant quadratic effect of time on adherence at high levels of locomotion, B = -1.61 *t*(222.98) = -2.24, *p* = .03. In order to better understand the quadratic trend, we examined the instantaneous rate of change in adherence scores at weeks 3 and 11—the midpoints of the first and second half of the study.

A significant positive rate of change in adherence scores at week 3 for high locomotors was found, B = 14.48, *t*(223.06) = 2.35, *p* = .02. However, high locomotors also exhibited a marginally negative rate of change in adherence scores at week 11, B = -11.34, *t*(223.07) = -1.79, *p* = .08. This suggests that healthy behaviors increased among high locomotors for the first half of the study, but their motivation began to diminish as the study continued. Even so, high locomotors likely benefit from accumulating a greater number of points throughout the entirety of the study. Indeed, locomotion was associated with marginally higher adherence scores at week 3 (B = 149.04, *t*(16.00) = 1.64, *p* = .12), significantly higher scores at week 7 (B = 193.64, *t*(16.09) = 2.13, *p* < .05), and marginally higher scores at week 11 (B = 169.93, *t*(15.71) = 1.88, *p* = .08). This suggests that even as individuals high in locomotion were trending toward lower adherence scores in the latter half of the study, they were still marginally outperforming those low in locomotion.

#### Conclusion

An exploration of adherence to healthy behavior found that individual differences in regulatory mode predicted distinct trends over time. In particular, individuals high in assessment trended toward generally improving their adherence over time in contrast to individuals low in assessment who trended toward showing decreases in adherence over time. Interestingly, unlike assessment, locomotion was associated with distinct quadratic patterns in adherence whereby high locomotion was associated with an upward trend in adherence during the first half of the study and a downward trend in the latter half, but still generally higher adherence than low locomotion. That assessment and locomotion were associated with different patterns in adherence suggest that greater investigation of regulatory mode in the context of mHealth behavioral intervention effectiveness is warranted. Notably, these results also pertain to self-reported healthy behavior, and these results would be magnified if these self-reported behaviors were associated with clinical benefits. As such, we next turned to investigating the relationship between total adherence over the course of the study and changes in HbA1C from baseline to post-intervention.

### Changes in HbA1C

Our mHealth intervention should improve clinical outcomes for diabetes patients to the extent that it motivates lifestyle changes in important domains like glucose tracking, exercise, medication adherence, and nutrition. We tested for an interaction between adherence, operationalized as total score earned through DiaSocial’s point system, and time on HbA1C levels. Total scores were standardized to enhance interpretation, time was dummy coded (0 = baseline, 1 = post-intervention), and treatment conditions (effects coded) and age (centered around the mean) were again included as covariates in these analyses. Time was modeled as a fixed effect, but intercepts were allowed to vary randomly. The two participants identified as having outlier HbA1C values were excluded from analyses, yielding a final sample size of 20. A comparison of analyses with different exclusion criteria is also reported in S3 Appendix.

**Fig 6:**
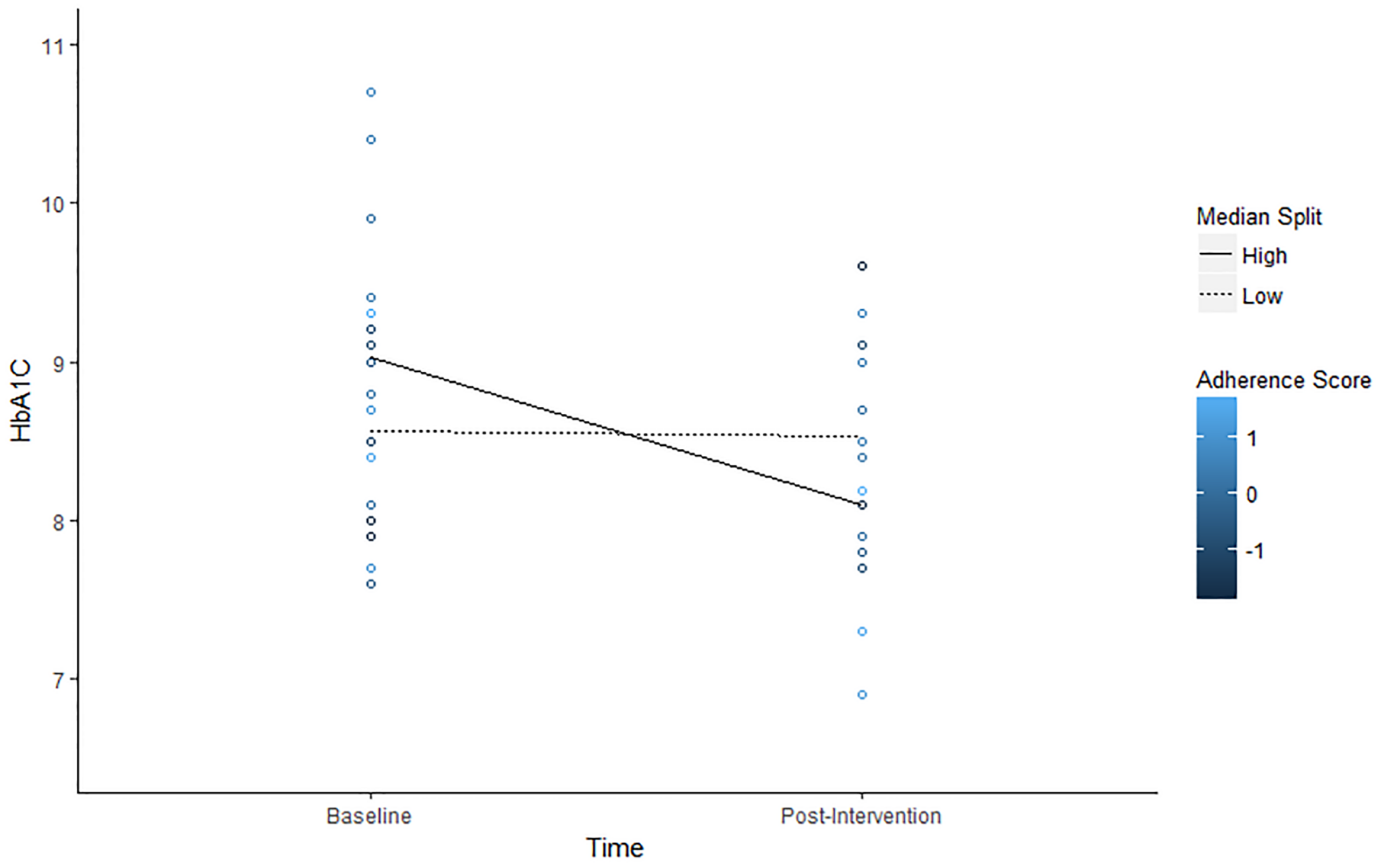
Observed values of HbA1C by individual adherence score. Colors represent standardized total adherence scores of participants and lines show average HbA1C levels for low (< 4954 total score) and high (> 4954 scale score) adherers as defined by a median split.

Table 5 reports descriptive statistics and correlations and Fig 6 depicts observed trends in the relationship between adherence scores and HbA1C over time. Table 6 reports results from the estimated model (and a plot of residual vs. predicted values presented in S1 Appendix). The linear mixed effects model was consistent witha significant effect of time reported in the table that can be interpreted as a significant decline in HbA1C at mean levels of total score, B = -0.48, *t*(18) = -2.95, *p* < .01. In addition, there was a non-significant effect of total score, indicating that there was no difference in baseline HbA1C between high and low scorers, B = 0.24, *t*(23.23) = 1.45, *p* = .16. Consistent with our prediction, there was a significant interaction effect of time and total score, B = -0.59, *t*(18) = -3.58, *p* < .01. Simple slopes analyses were conducted to further decompose the interaction.

**Table 5:** Descriptive statistics and Bivariate correlations (N = 20).

	*M*	*SD*	1	2	3	4	5	6
1. Age	68.25	6.19	‒					
2. Locomotion	3.77	0.52	-.43 ^ǂ^	‒				
3. Assessment	2.84	0.71	.08	.06	‒			
4. Days App Used	66.25	24.84	-.01	.37	.26	‒		
5. Total Adherence Score	4537.75	2355.43	-.17	.46*	.16	.86**	‒	
6. Baseline HbA1c	8.80	0.88	-.28	.42^ǂ^	.22	.44^ǂ^		‒
7. Post HbA1C	8.31	0.75	.04	-.25	-.15	-.29	-.45^ǂ^	.36

^ǂ^ *p* < .10.

*p < .05.

** *p* < .001.

**Table 6:** Model estimates predicting HbA1C.

	Est.	SE	*t*	*p*
*Fixed Effects*				
Intercept	8.79	0.16	54.92	< .001
Age	-0.04	0.02	-1.83	.09
T2	0.77	0.25	3.06	< .01
T3	-0.20	0.23	-0.89	.39
T4	-0.25	0.22	-1.11	.28
Time	-0.48	0.16	-2.98	< .01
Adherence	0.23	0.16	1.45	.16
Time x Adherence	-0.59	0.16	-3.58	< .01
*Random Effects*				
σ^2^	.26	.09		< .01
τ_00_	.22	.14		.12

σ^2^ = residual, τ_00_ = variance in intercept by participant.

**Fig 7:**
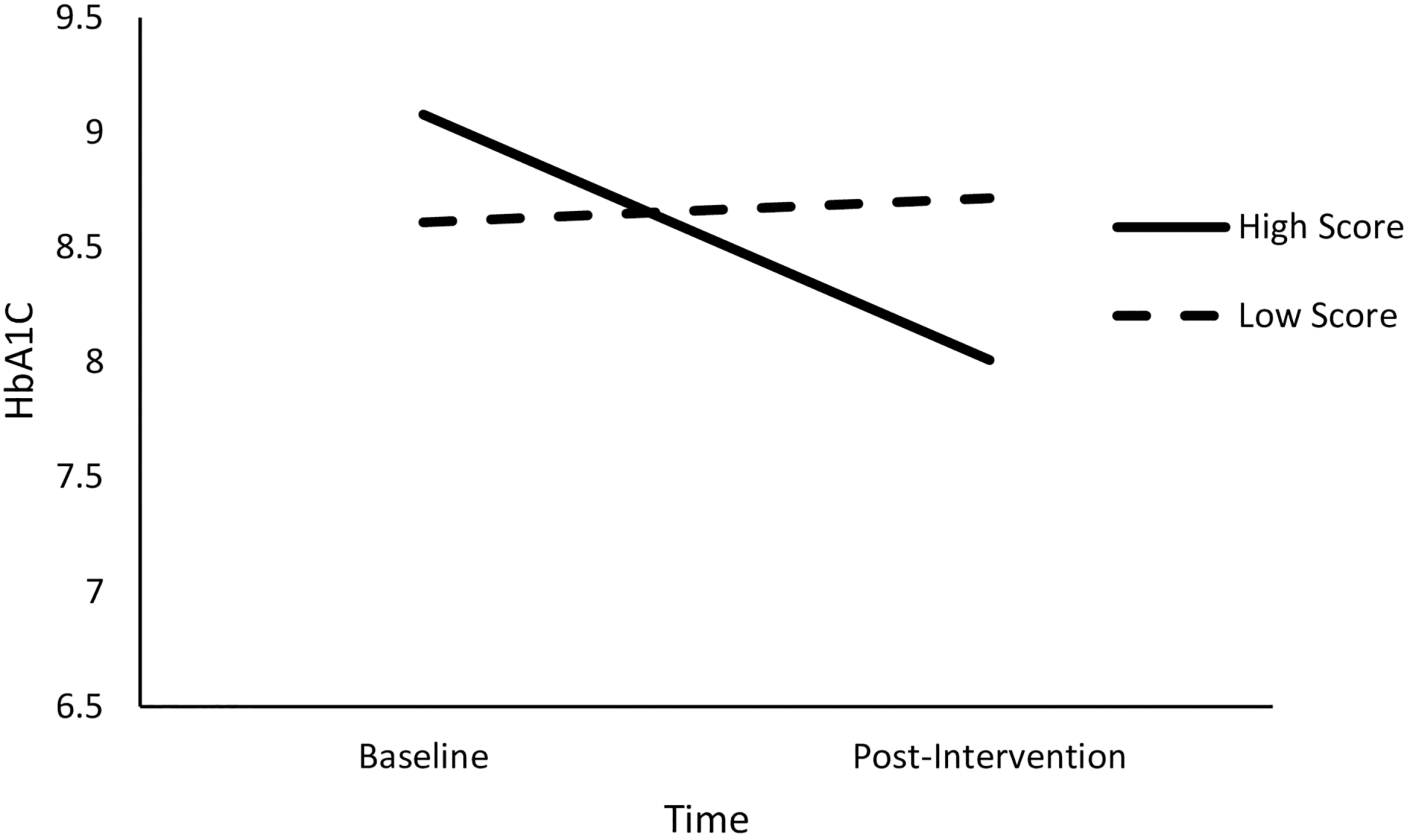
Change in predicted HbA1C over time as a function of total adherence scores.

Results of the simple slopes analyses included a non-significant relationship between time and HbA1C at low (-1 SD) total scores, B = 0.11, *t*(18) = 0.46, *p* = .65. In contrast, time was significantly associated with lower A1C levels when app usage was high, B = -1.07, *t*(18) = -4.64, *p* < .001. The above findings are depicted in Fig 7. These results suggest that participants who exhibited strong adherence, as indicated by self-reported diet, exercise, medication, and glucose monitoring, showed about a 1.0 point drop in HbA1C from baseline to post-intervention on average, controlling for age and treatment condition. In contrast, those who exhibited mean levels of adherence showed less of a decline in HbA1C, at about .48 points on average. Finally, individuals who exhibited low adherence showed no change in baseline and post-intervention HbA1C.

## Discussion

The primary objective of this pilot study was to explore potential individual differences in the effectiveness of an mHealth intervention to improve health behaviors and clinical outcomes among older adults with diabetes. We argued that regulatory mode, i.e., the degree to which an individual was a locomotor or an assessor, would moderate the effects of an intervention that required goal setting and included gamification. While we found only marginal relationships between assessment and adherence, our results were consistent with the prediction that effectiveness of the intervention is conditional on locomotion. Our pilot study provided preliminary evidence that implementation of an mHealth behavioral intervention would differentially impact treatment adherence according to individual differences in regulatory mode, and treatment adherence was further associated with improvements in levels of HbA1C.

Our research extends the literature on the use of mHealth tools to improve diabetes management[44–46] by emphasizing the role of personality differences. An important implication that follows is the need for greater attention to the interplay between personality and mHealth interventions to better understand their effectiveness. Consistent with our expectation that the app’s use of goal-setting would be a good fit with locomotors’ eagerness to engage in goal-oriented movement[35,36], locomotion was associated with greater overall treatment adherence. Interestingly, high locomotors also exhibited a quadratic pattern in adherence—while scores were high overall for locomotors, they increased from the beginning to the mid-point of the study before beginning to trend downwards.

Evidence that locomotors like to multitask[47] suggest one possible reason why locomotors might begin to show a decline in adherence—a propensity to get bored more easily. Future interventions could try to mitigate this by introducing novel goals and challenges or levels throughout the intervention period to maintain locomotors’ interest. These findings support the argument that mHealth tools can serve as a catalyst for locomotors to direct their energy toward better disease management when they narrow focus to specific goals with features like gamification.

In contrast to our findings related to locomotion, the association between assessment and adherence was less clear. Although linear trends in adherence over time were found to depend on individual differences in assessment, the positive trend for high assessors, who we predicted would enjoy the self-monitoring features of our mHealth app, did not reach significance (nor the negative trend for low assessors). Thus, it is difficult to infer whether these findings are inconsistent with evidence showing benefits for a “fit” between achievement contexts and an individual’s level of assessment[41] and to what extent. However, we argue that these findings, along with support for the moderating role of locomotion, highlight the promise in theory-driven investigation of fit between personality and mHealth interventions and underscore the need for more research along these lines.

Our pilot study also offered initial support for the validity of our gamification system in which we allocated points for different health behaviors. With greater amounts of points being associated with greater declines in HbA1C, we have encouraging evidence that our point system is incentivizing achievable, clinically important health behaviors. The association between points and improved clinical outcomes also suggests that most individuals are honest and accurate in their reporting even though past research has identified shortcomings of self-report measures of nutrition[48] and physical activity[49,50].

### Limitations and future directions

In our pilot, we also explored the effectiveness of different app features including communication with provider and peer teams. Overall, treatment effects were not supported, but this was not unexpected given some of the limitations of our study, which bear further elaboration. Most notably, the sample size was small, meaning the study was underpowered. Though the large number of observations per participant mitigated the small sample size to some extent, our ability to detect differences across treatment conditions was limited and we did not have the power to explore interactions between individual differences in regulatory mode and the effectiveness of specific treatments. In addition, because the communication features in the app were rudimentary and not heavily used during the study, we did not expect significant differences across conditions in the pilot. Favorably, the app design enabled us to collect intensive longitudinal data on each participant, allowing us to make the most of our smaller sample size when examining trends in self-reported adherence behavior. Unfortunately, HbA1C levels were also assessed for each patient at different times, depending on their regular patient care schedule, thereby introducing some noise in our ability to detect differences in clinical outcomes. In the next trial testing the effectiveness of DiaSocial (or a similar mHealth app), we will seek to expand our sample size, refine within-app messaging, and have more consistent clinical measures to address these limitations.

It should be noted that our intervention was delivered via a tablet rather than smartphone, which might be a better fit for older adults[13]. Reinforcing other mHealth research with older adults[51,52], our pilot study suggests that the use of mHealth tools to improve management of chronic diseases in older adults is highly feasible, but additional research should test for differences in delivery by tablets and smartphones, and for differences in effectiveness according to individual health and technology literacy. Our sample also presents limitations to the generalizability of our findings. More specifically, most of our participants were white male veterans, which is consistent with other research with veteran samples[53], but additional research with more balanced ratios of men, women and races is needed to explore how regulatory mode contributes to mHealth success among women and different cultures. This is especially true given some evidence of gender differences in effectiveness of lifestyle interventions among veterans, with women benefitting more than men[54].

Importantly, our findings also raise questions about how to craft interventions that would benefit low locomotors and assessors. For example, future research could explore implementing manipulations of regulatory mode that have been used in laboratory-based research[55] through messages delivered via the app. Manipulating regulatory mode in this manner might enable even those typically low in locomotion and assessment to capitalize on the intervention. It will also be critical to explore how healthcare organizations and professionals can adopt and operationalize tailored mHealth through implementation science research.

### Conclusion

The widespread use of mobile technology, cost-effective cloud computing infrastructure and broad cellular network coverage in the U.S. makes mHealth a promising and pragmatic candidate for helping patients with diabetes improve their diabetes self-management. Overall, the relatively high engagement levels seen in our pilot are encouraging and suggest that mHealth tools are a viable medium to deliver chronic disease interventions and to monitor self-care among older adults, consistent with evidence that older adults are indeed interested in using mHealth technologies[14,15,51,52]. Our pilot study provides initial evidence of individual differences in the effectiveness of an mHealth tool, offering an important contribution to the mHealth literature that is guided by psychological theory. In order to leverage the advantages of mHealth tools for all people, we must continue developing our understanding of what types of designs will be motivating to different people. A failure to do so will blunt the impact of mHealth interventions and leave some individuals behind.

## Supporting information

S1 File Supporting information. CONSORT Checklist.
(PDF)

S2 File Supporting information. Study protocol.
(PDF)

S1 Appendix Supporting information. Tables providing additional information about HbA1c measures, descriptive statistics, and a breakdown of the app’s point system.
(PDF)

S2 Appendix Supplementary analyses. Tables reporting supplementary analyses specific to glucose, nutrition, exercise, and medication adherence scores.
(PDF)

S3 Appendix Supplementary analyses. Tables reporting analyses of HbA1C using different exclusion criteria as a robustness check.
(PDF)
