# Supplementary material for: Individual differences in regulatory mode moderate the effectiveness of a pilot mHealth trial for diabetes management among older veterans
Source: PLoS One. 2018 Mar 7;13(3):e0192807. doi: 10.1371/journal.pone.0192807 (PMC5841664; doi:10.1371/journal.pone.0192807)
Supplement: S2 File — Study protocol. (PDF) [file pone.0192807.s002.pdf]

Study Protocol

In this study, the investigators propose to design and test a motivation psychology-based smart engagement system (MOSES), which is a software application on a digital tablet device. The pre-loaded tablets will be provided to adults with T2DM (age 60+ with high glucose). The software will allow the patients to record diabetes self-care activities (exercise, glucose, nutrition, medication adherence), pursue goals, support communication between the patient and a health coach, support communication between peer patients, and visualize health status more easily by patients and providers. This research program will enroll 88 patients (4 intervention groups of 12 persons each and a 40-person control group) in a 90-day pilot study to test and refine the design of the application and its effectiveness in supporting care plan goals.

Primary Aim 1: Design, implement, and optimize a motivation psychology-based smart engagement system (MOSES) for older adults with diabetes.

Secondary Aim 2: Determine if providing older adult diabetic patients to care managers and peers via a digital tablet-based software application leads to improved diabetes management as measured by blood glucose control.

Secondary Aim 3: Refine the personality attributes used to tailor the interaction with the application.

The investigators propose to conduct a 90-day pilot study to establish the effects and value of MOSES in the management of diabetes. The pilot study will provide initial data to answer the following research questions: (1) What are the effects of MOSES technology on older adults' diabetes self-care? (2) What type of social engagement intervention is more effective for what aspects of diabetes care? (3) What personality characteristics predict older adults' diabetes self-care?; (4) What personality characteristics moderate the effectiveness of mobile device (esp. tablet-based) social engagement interventions on older adults' diabetic care? (5) What model can be developed from our data to predict probability of effective response to interventions tailored to individual patients, e.g. by message type for clinical, usage and personality attributes?

Subjects Older adults are the target population for this study. Adult volunteers age 60 and above with an HbA1C of >7.9% and having the capacity to provide informed consent will be eligible to participate. Participants will be randomized to usual care or to an intervention group (described below). Once enrolled in the study and randomized to either the intervention or the control group, the Research Certified Diabetes Educator (CDE) will review all participants' clinical data and assist with providing feedback messages to the participants regarding their self-care progress. The Research CDE will alert physicians to any clinical data that would warrant a change in clinical management (e.g. hyper or hypoglycemia).

Manipulations

There are four manipulations in the experiment: (1) patient recording i.e. using the tablet and app to track and review progress (this increases mindfulness, engagement, activation, and informedness, thereby leading to healthier behaviors than the control condition), (2) patient recording with physician involvement via Health-feed communication messages (in addition to the benefits listed above, this provides expert feedback from a trusted source on actions, helps with goal setting, provides motivational reinforcement of positive behavior and sanction against negative, etc.), (3) patient recording with peer interaction i.e. using Health-feed messages, Community Views and Health Tournaments (in addition to the benefits listed for (1), peer effects have been observed in a variety of different settings such as educational attainment). People tend to emulate the behavior of peers, setting in motion a cycle of group conformism. People may also be motivated by a desire to compete in a peer setting, and (4) patient recording with peer interaction AND physician involvement. This final group will permit analysis of the interaction effects of the two forms of social engagement. Thus, the experiment will be done with the following groups:

- Group A: the control group, usual care, (n=40)
- Group B: individual patients using app w/o provider support (n=12)
- Group C: individual patients using app w/ provider support (n=12)
- Group D: peer patient teams using app w/o provider support (n=12; 4 teams)
- Group E: peer patient teams using app with provider support (n=12; 4 teams)

Groups will be assigned to achieve age, gender and HbA1C matching at baseline. For groups with provider engagement (E), the provider (endocrinologist/CDE) will provide initial advice to the subjects, as well as continuous monitoring. The study will require a dedicated diabetes educator, who will spend 2 hours a day to keep in touch with the subjects, including reviewing the activity logs, sending reminder and motivational messages, as well as moderating the discussions in the peer engagement group. The CDE will triage the text messages from subjects and trigger the physician engagement when necessary. The study CDE will review participant data routinely and will maintain communication with the study team on a timely basis.

During the experiments, adverse events will be closely monitored by the physician and CDE, as well as through subject self-reports. The MOSES app has a built-in function to highlight potential adverse effects in the activity list and physician to-do list. The investigators will also conduct a weekly review of subject activity log.

Planned Analyses

Beyond the group fixed effects, regression analyses will be performed to test hypotheses about the role of personality across different diabetes management outcomes. Analyses pertaining to

research questions about personality will include all conditions of the experiment.
