# Supplementary material for: Individual differences in regulatory mode moderate the effectiveness of a pilot mHealth trial for diabetes management among older veterans
Source: PLoS One. 2018 Mar 7;13(3):e0192807. doi: 10.1371/journal.pone.0192807 (PMC5841664; doi:10.1371/journal.pone.0192807)
Supplement: S1 Appendix — Tables providing additional information about HbA1c measures, descriptive statistics, and a breakdown of the app’s point system. (PDF) [file pone.0192807.s003.pdf]

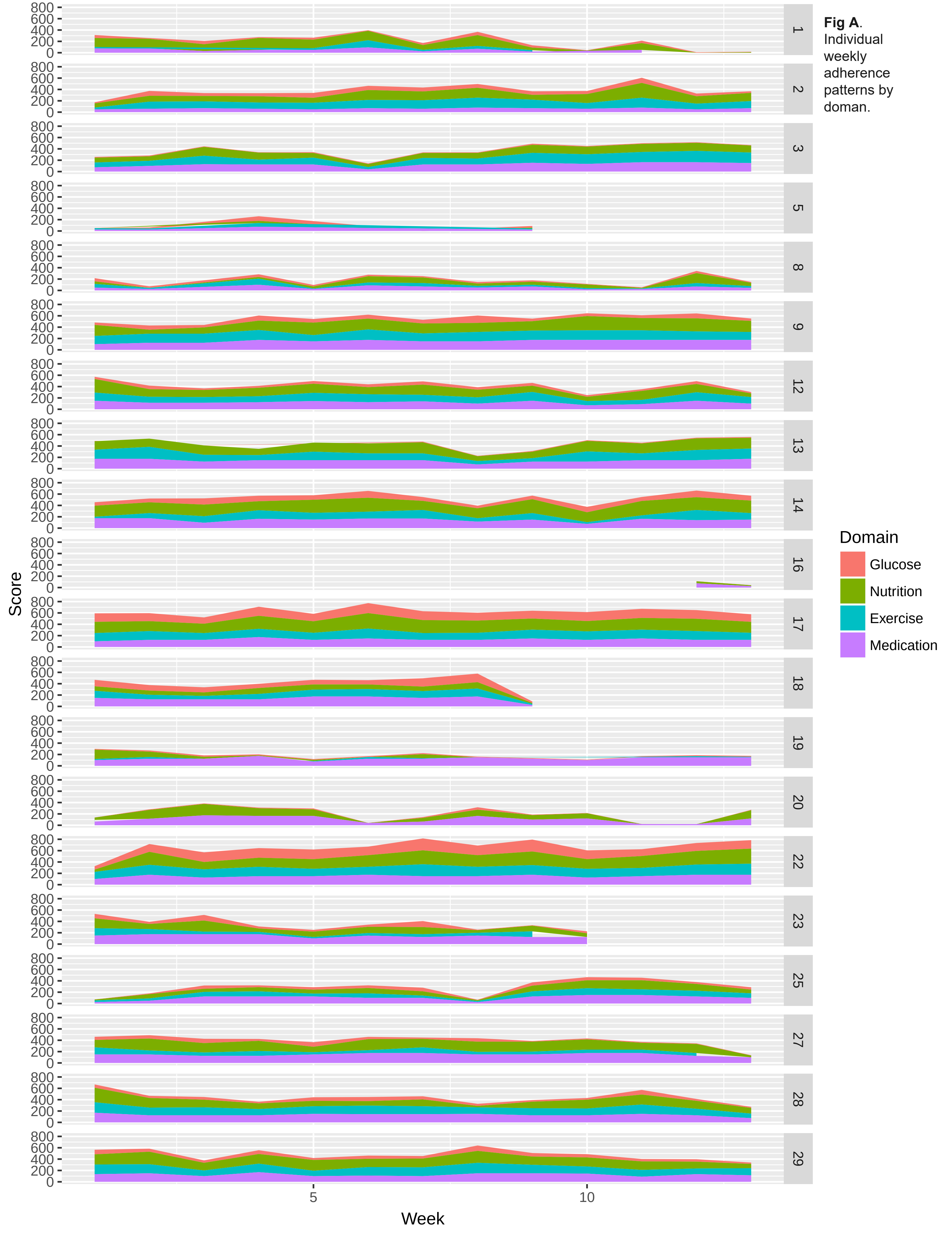

Fig A. Individual weekly adherence patterns by doman.

Table A. Summary of HbA1C Collection Information

	Baseline Measure		Post Measure	
ID	Value (%)	Date from Start	Value (%)	Date from Start
1	9.2	-2	7.7	+74
2	8.1	-2	7.9	+97
3	9.9	-72	8.7	+26
5	7.9	+20	8.1	+62
8	8.5	+6	8.2	+91
9	8.7	-64	6.9	+91
11	9.4	-63	14.0	+104
12	9.4	+19	8.2	+105
13	10.4	-97	8.5	+62
14	7.7	+6	7.3	+82
15	11.6	-57	13.0	+111
16	8.0	-56	9.6	+72
17	8.4	-57	7.3	+34
18	9.0	-57	8.4	+83
19	9.1	-127	9.1	+104
20	8.8	-50	9.6	+103
22	9.3	-26	8.2	+62
23	7.6	-15	8.1	+91
25	7.6	-14	7.8	+105
27	8.8	-9	8.4	+71
28	8.8	-44	9.0	+55
29	10.7	-19	9.3	+89

Negative values indicate that the measure was taken the stated number of days *before* the start of the study. Positive values indicate that the measure was taken *after* the start of the study. Baseline HbA1C measures taken on or before the start of the study period would have values <=0. Post-study HbA1C measures assessed on the last day of or after the study would have values >= +91.

Table B. Allocation of Points for Healthy Behavior

Behavior	Value
Glucose Maximum Daily Total	*30 points*
Report glucose twice	5 points
Report glucose three times	7 points
Report glucose four times	10 points
Maintain glucose within target range for half of the recordings	10 points
Maintain glucose within target range for all recordings	20 points
Nutrition Maximum Daily Total	*45 points*
Report 2 meals or snacks	10 points
Report 3 meals or snacks	15 points
Report 4 meals or snacks	4 points
Report 5 meals or snacks	5 points
Report 6 meals or snacks	6 points
Keep carbs at or below consumption goal	20 points
Eat 3 servings of fruits or vegetables	10 points
Exercise Maximum Daily Total	*35 points*
Report exercise or sync with Fitbit	5 points
Engage in 15 minutes of exercise	20 points
Engage in 30 minutes of exercise	25 points
Engage in 45 minutes or more of exercise	30 points
Medication Maximum Daily Total	*30 points*
Reported medication information	10 points
Took all recommended medications that day	20 points
Took some, but not all recommended medications that day	10 points

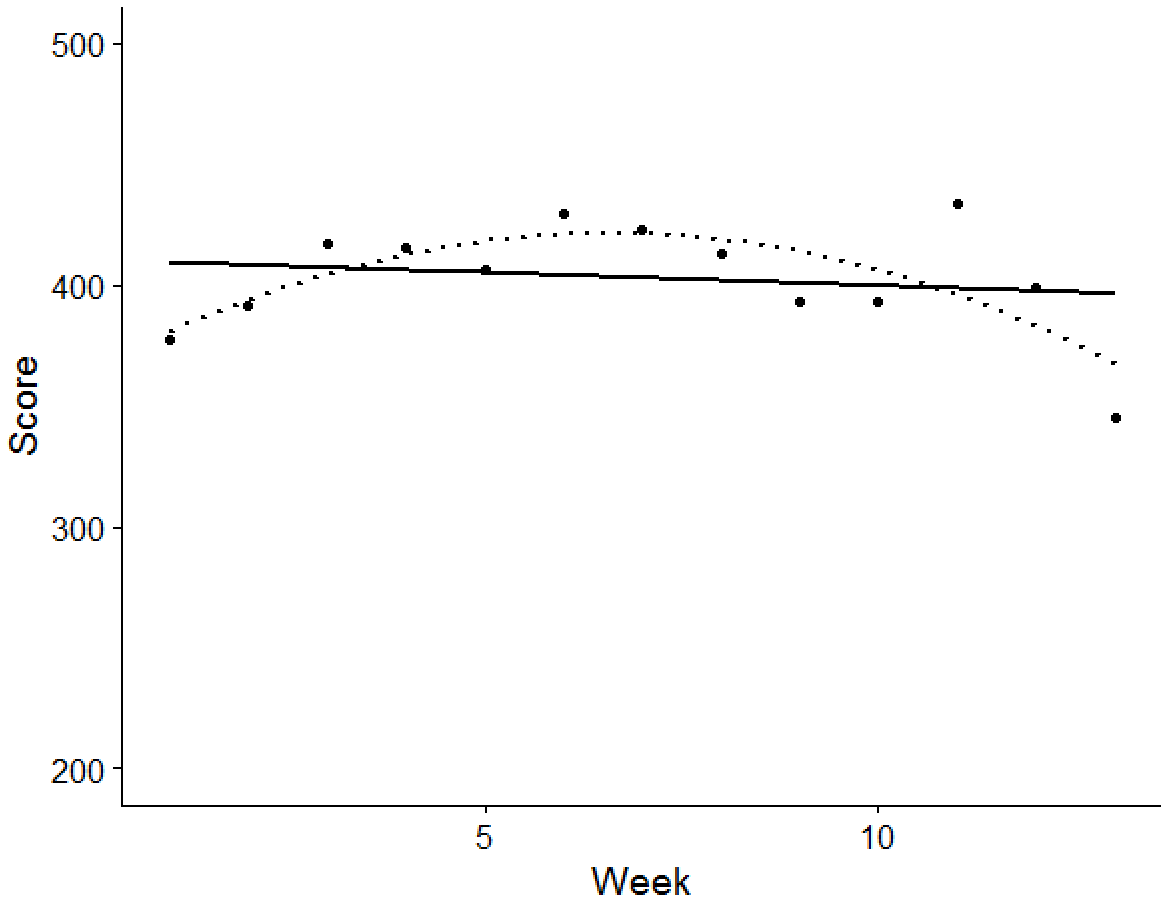

Fig B. Depicts mean levels of weekly adherence with superimposed linear and quadratic trends.

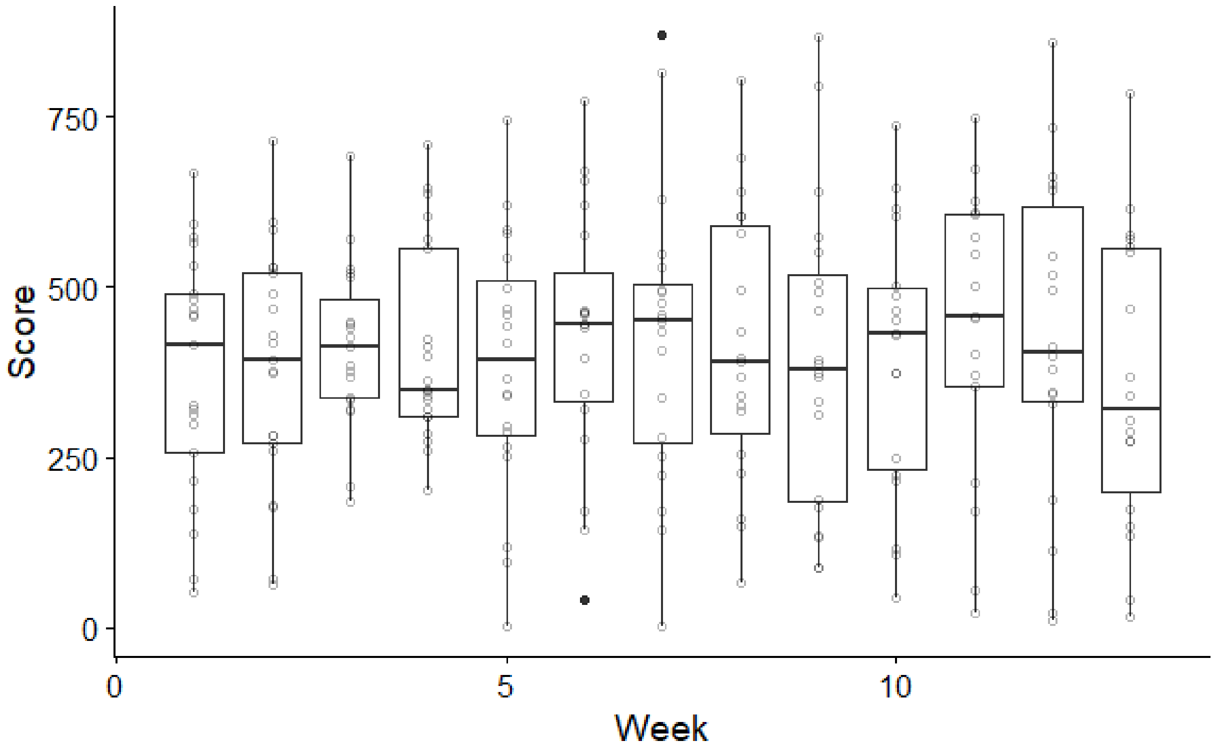

Fig C. Depicts boxplot of weekly adherence scores.

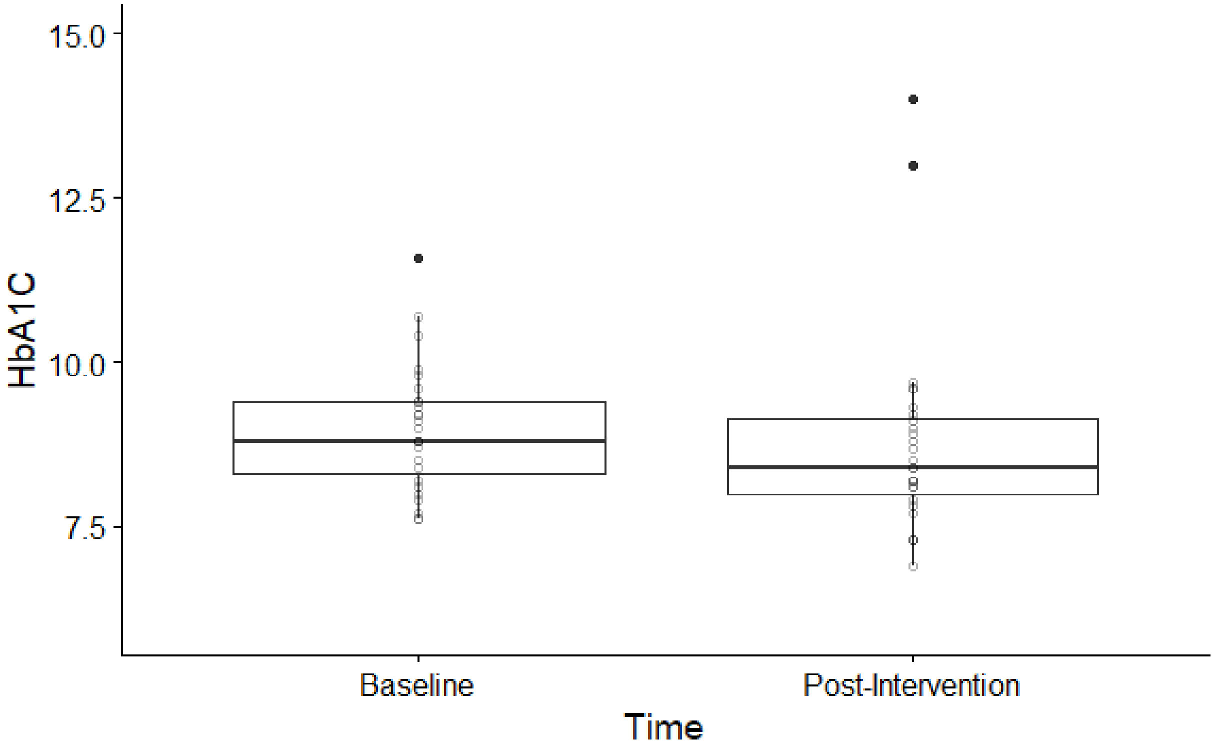

Fig D. Depicts boxplot of HbA1C values by time.

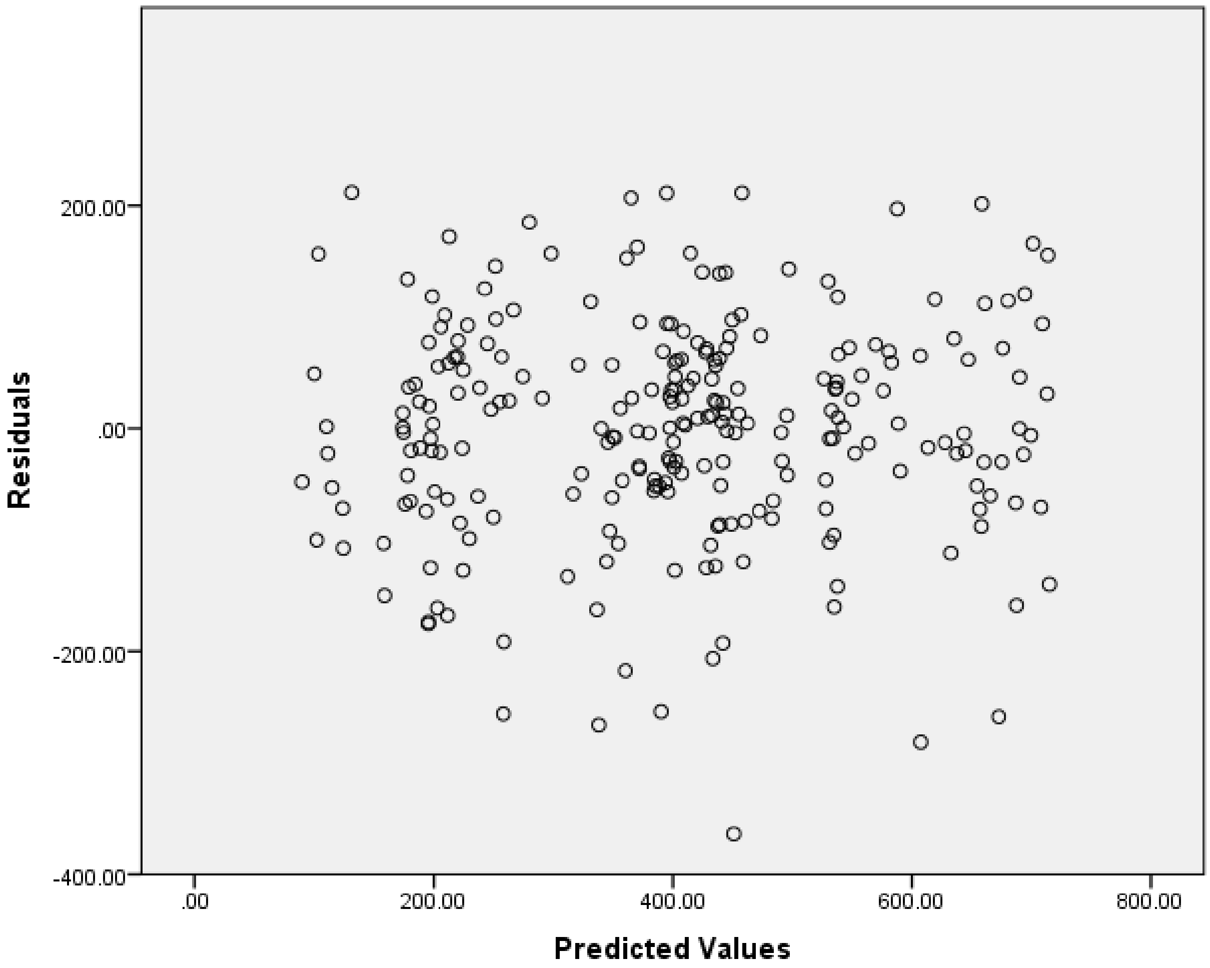

Fig E. Depicts diagnostics of the mixed effects model examining trends in weekly adherence.

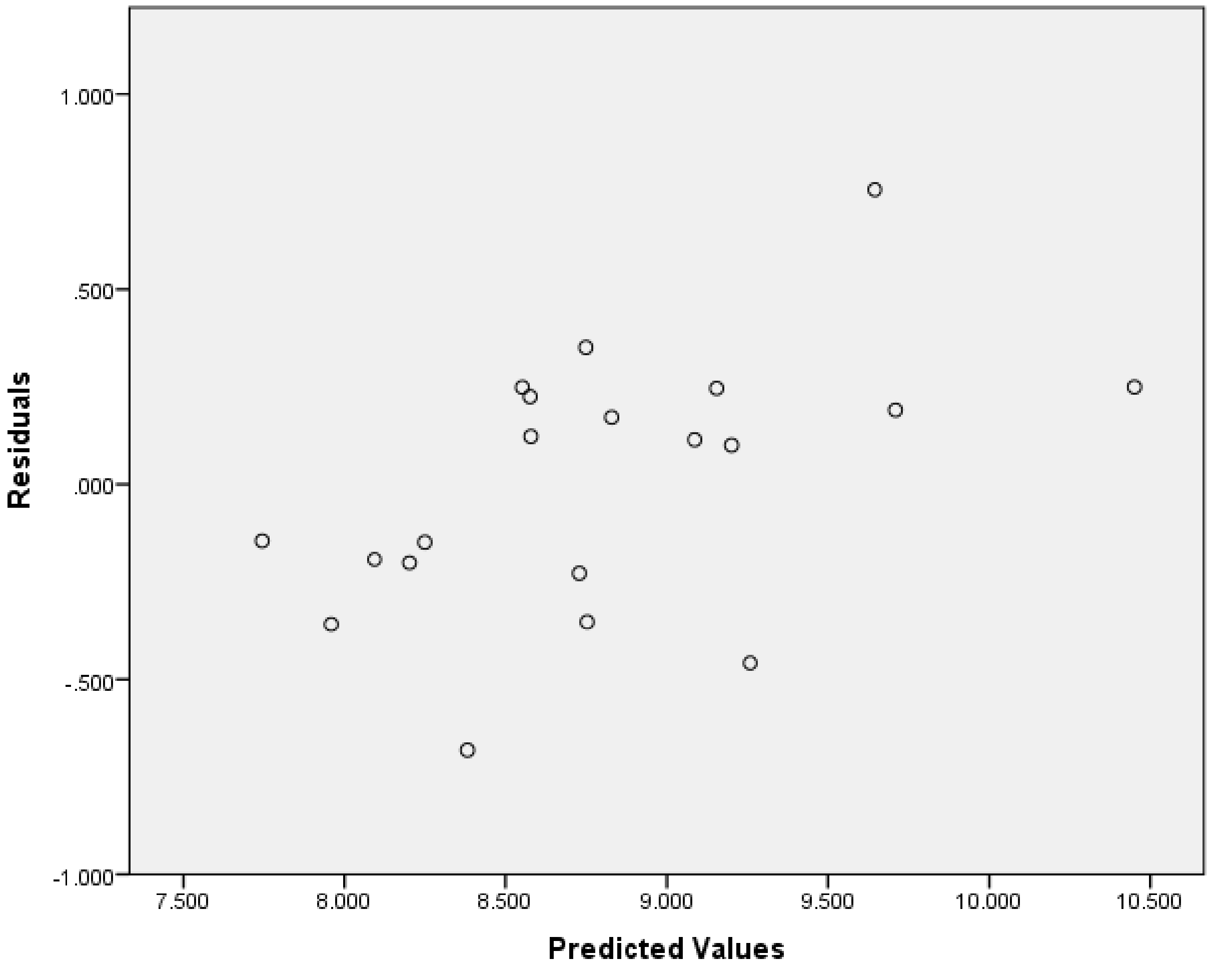

Fig F. Depicts diagnostics of the mixed effects model examining changes in HbA1C.
