# Supplementary material for: Individual differences in regulatory mode moderate the effectiveness of a pilot mHealth trial for diabetes management among older veterans
Source: PLoS One. 2018 Mar 7;13(3):e0192807. doi: 10.1371/journal.pone.0192807 (PMC5841664; doi:10.1371/journal.pone.0192807)
Supplement: S2 Appendix — Tables reporting supplementary analyses specific to glucose, nutrition, exercise, and medication adherence scores. (PDF) [file pone.0192807.s004.pdf]

Modeling of Between-Group Effects on Changes in HbA1C

Fixed effects

Both time (0 = baseline, 1 = post-intervention) and treatment condition were dummy coded. Measures of HbA1C levels were obtained for all participants, including those in the control condition, which allowed us to include observations from all five arms of the study. Therefore, there was a natural comparison group against which we wanted to assess treatment effects and analyses were conducted with dummy coding. That is, the effects of T1-T4 were contrasted with Control (C) with a dummy coding procedure. In order to test for differences in the rate of change of HbA1C between treatment conditions, we included interaction effects of time and each treatment condition contrast in the model. The interaction effects of time and treatment condition were of central interest in these analyses, as they would reveal whether rates of change in HbA1C differed across treatments. Age was again included as a covariate in these analyses and centered around its mean.

Random effects

Intercepts were allowed to vary randomly for each participant, but all other effects were treated as fixed and the covariance structure consisted of a scaled identity matrix.

Final Model and Results

Our model can be represented with the following equations:

$$HbA1C_{ij} = \beta_{0j} + \beta_{1j}(Time_{ij}) + r_{ij} \tag{1}$$

$$\beta_{0j} = \gamma_{00} + \gamma_{01}(T1_j) + \gamma_{02}(T2_j) + \gamma_{03}(T3_j) + \gamma_{04}(T4_j) + \gamma_{05}(Age_j) + u_{0j} \tag{2}$$

$$\beta_{1j} = \gamma_{10} + \gamma_{11}(T1_j) + \gamma_{12}(T2_j) + \gamma_{13}(T3_j) + \gamma_{14}(T4_j) \tag{3}$$

Modeling of Regulatory Mode Effects on Trends in Adherence

Fixed Effects

Time was treated as a continuous variable in our analyses. As already noted, we expected that weekly adherence might exhibit a quadratic trend: participants experienced a learning and success-building period reflected by a linear increase in adherence over the first half of the study, and followed by a period of stagnation and possible decline as the novelty of the intervention waned in the later weeks. Thus, both linear and quadratic trends of time were modeled and time was centered around the midpoint of the treatment duration (week 7 = 0) to enhance interpretability. In order to test the moderating role of regulatory mode, the model included interaction effects of time with locomotion and time with assessment. Both locomotion and assessment were treated as continuous variables and centered around their means.

The model also controlled for several covariates. Despite not finding significant relationships with overall adherence, we controlled for treatment effects by including effects coded contrasts in the model where T1 served as the reference group. In addition, we controlled for age, which was centered around its mean.

Random Effects

Intercepts were allowed to vary randomly for each participant, but all other effects were treated as fixed and the covariance structure consisted of a scaled identity matrix.

Final Model and Results

The tested model can be represented by the below equations:

$$Adherence\ Score_{ij} = \beta_{0j} + \beta_{1j}(Time_{ij}) + \beta_{2j}(TimeSQ_{ij}) + r_{ij} \qquad (4)$$

$$\beta_{0j} = \gamma_{00} + \gamma_{01}(Locomotion_j) + \gamma_{02}(Assessment_j) + \gamma_{03}(T2_j) + \gamma_{04}(T3) + \gamma_{05}(T4_j) + \gamma_{06}(Age_j) + u_{0j} \qquad (5)$$

$$\beta_{1j} = \gamma_{10} + \gamma_{11}(Locomotion_j) + \gamma_{12}(Assessment_j) \quad (6)$$

$$\beta_{2j} = \gamma_{20} + \gamma_{21}(Locomotion_j) + \gamma_{22}(Assessment_j) \quad (7)$$

Table A. Model Estimates Predicting Weekly Glucose Adherence Scores

	Est.	SE	*t*	*p*
Fixed Effects				
Intercept	57.55	9.78	5.89	< .001
Age	0.49	2.05	0.24	.81
T2	-27.42	19.19	-1.43	.17
T3	9.91	16.54	0.60	.56
T4	14.46	16.67	0.87	.40
Time	-0.38	0.40	-0.97	.33
Locomotion	54.61	23.30	2.34	.03
Assessment	-14.64	14.67	-1.00	.33
$Time^2$	-0.38	0.12	-3.27	< .001
Time x Locomotion	0.12	0.77	0.16	.87
Time x Assessment	-0.09	0.63	-0.14	.89
$Time^2$ x Locomotion	-0.32	0.23	-1.42	.16
$Time^2$ x Assessment	0.11	0.18	0.60	.55
Random Effects				
σ^2	495.15	47.32		< .001
τ_{00}	19599.86	735.68		< .01

σ^2 = residual, τ_{00} = variance in intercept by participant

Table B. Model Estimates Predicting Weekly Nutrition Adherence Scores

	Est.	SE	*t*	*p*
Fixed Effects				
Intercept	125.85	12.77	9.86	< .001
Age	0.39	2.62	0.15	.88
T2	-21.49	24.29	-0.88	.39
T3	-11.81	21.14	-0.56	.59
T4	19.59	21.24	0.92	.37
Time	0.01	0.82	0.01	.99
Locomotion	79.58	30.34	2.62	.02
Assessment	-8.68	19.28	-0.45	.66
$Time^2$	-0.03	0.24	-0.14	.89
Time x Locomotion	1.32	1.60	0.82	.41
Time x Assessment	2.44	1.32	1.84	.07
$Time^2$ x Locomotion	-1.14	0.48	-2.40	.02
$Time^2$ x Assessment	0.24	0.38	0.63	.53
Random Effects				
σ^2	2176.51	207.53		< .001
τ_{00}	3028.05	1205.34		.01

σ^2 = residual, τ_{00} = variance in intercept by participant

Table C. Model Estimates Predicting Weekly Exercise Adherence Scores

	Est.	SE	*t*	*p*
Fixed Effects				
Intercept	96.77	11.35	8.53	< .001
Age	-1.85	2.52	-0.74	.47
T2	1.78	20.64	0.09	.93
T3	-5.38	19.22	-0.28	.78
T4	6.02	18.55	0.33	.75
Time	1.00	.62	1.61	.11
Locomotion	47.04	27.50	1.71	.11
Assessment	10.48	17.16	0.61	.55
$Time^2$	.09	.18	0.50	.61
Time x Locomotion	0.91	1.20	0.76	.45
Time x Assessment	2.47	0.98	2.52	.01
$Time^2$ x Locomotion	-0.64	.35	-1.81	.07
$Time^2$ x Assessment	0.37	0.28	1.32	.19
Random Effects				
σ^2	1069.89	108.91		< .001
τ_{00}	2193.20	913.55		.02

σ^2 = residual, τ_{00} = variance in intercept by participant

Table D. Model Estimates Predicting Weekly Medication Adherence Scores

	Est.	SE	*t*	*p*
Fixed Effects				
Intercept	119.42	8.76	13.64	< .001
Age	-0.40	1.80	-0.22	.83
T2	-6.23	16.70	-0.37	.72
T3	5.59	14.52	0.39	.71
T4	9.60	14.59	0.67	.52
Time	0.21	0.54	0.38	.70
Locomotion	-0.43	20.79	-.02	.98
Assessment	10.21	13.21	.77	.45
$Time^2$	-.23	.16	-1.44	.15
Time x Locomotion	.30	1.06	0.29	.78
Time x Assessment	2.19	0.89	2.46	.02
$Time^2$ x Locomotion	0.08	0.32	0.25	.80
$Time^2$ x Assessment	0.28	0.25	1.09	.28
Random Effects				
σ^2	935.06	89.69		< .001
τ_{00}	1438.33	576.88		.01

σ^2 = residual, τ_{00} = variance in intercept by participant
