# Supplementary material for: Individual differences in regulatory mode moderate the effectiveness of a pilot mHealth trial for diabetes management among older veterans
Source: PLoS One. 2018 Mar 7;13(3):e0192807. doi: 10.1371/journal.pone.0192807 (PMC5841664; doi:10.1371/journal.pone.0192807)
Supplement: S3 Appendix — Tables reporting analyses of HbA1C using different exclusion criteria as a robustness check. (PDF) [file pone.0192807.s005.pdf]

Table A. Model Estimates Predicting HbA1C Including Outliers (N = 22)

	Est.	SE	*t*	*p*
Fixed Effects				
Intercept	8.98	0.28	32.38	< .001
Age	-.06	0.04	-1.33	.20
T2	0.48	0.46	1.04	.31
T3	-0.57	0.41	-1.39	.18
T4	-0.64	0.41	-1.57	.14
Time	-0.27	0.28	-0.59	.56
Adherence	0.41	0.28	1.46	.16
Time x Adherence	-0.65	0.28	-2.32	.03
Random Effects				
σ^2	.85	.27		< .01
τ_{00}	.82	.46		.07

σ^2 = residual, τ_{00} = variance in intercept by participant. *Note*. The same multilevel regression models explaining changes in HbA1C levels were tested with different exclusion criteria for the analyses, with the results excluding only outliers reported in the main text.

Table B. Model Estimates Predicting HbA1C Excluding Early HbA1C Measures (N = 17)

	Est.	SE	*t*	*p*
Fixed Effects				
Intercept	8.96	0.37	24.20	< .001
Age	-0.07	0.05	-1.26	.23
T2	0.73	0.69	1.07	.31
T3	-0.59	0.51	-1.16	.27
T4	-0.84	0.67	-1.26	.24
Time	-0.08	0.35	-0.22	.83
Adherence	0.62	0.40	1.57	.14
Time x Adherence	-0.76	0.37	-2.08	.06
Random Effects				
σ^2	1.06	.39		< .01
τ_{00}	1.09	.72		.13

σ^2 = residual, τ_{00} = variance in intercept by participant. *Note*. Participants excluded for timing of their A1C measurements included anyone whose baseline assessment took place over 100 days before study start (participant 19) or whose post-study A1C measure was taken less than 60 days after study start (participants 3, 17, 28). Participant 5 was also excluded for having only 42 days between their baseline and post-study measures.
